# HIV-specific Fc effector function early in infection predicts the development of broadly neutralizing antibodies

**DOI:** 10.1371/journal.ppat.1006987

**Published:** 2018-04-09

**Authors:** Simone I. Richardson, Amy W. Chung, Harini Natarajan, Batsirai Mabvakure, Nonhlanhla N. Mkhize, Nigel Garrett, Salim Abdool Karim, Penny L. Moore, Margaret E. Ackerman, Galit Alter, Lynn Morris

**Affiliations:** 1 Centre for HIV and STI’s, National Institute for Communicable Diseases, Johannesburg, Gauteng, South Africa; 2 Faculty of Health Sciences, University of the Witwatersrand, Johannesburg, Gauteng, South Africa; 3 Department of Microbiology and Immunology, Peter Doherty Institute for Infection and Immunity, The University of Melbourne, Parkville, Victoria, Australia; 4 Thayer School of Engineering, Dartmouth College, Hanover, New Hampshire, United States of America; 5 Centre for the AIDS Programme of Research in South Africa (CAPRISA), University of KwaZulu-Natal, Durban, KwaZulu Natal, South Africa; 6 Ragon Institute of MGH, MIT and Harvard, Cambridge, Massachusetts, United States of America; Vaccine Research Center, UNITED STATES

## Abstract

While the induction of broadly neutralizing antibodies (bNAbs) is a major goal of HIV vaccination strategies, there is mounting evidence to suggest that antibodies with Fc effector function also contribute to protection against HIV infection. Here we investigated Fc effector functionality of HIV-specific IgG plasma antibodies over 3 years of infection in 23 individuals, 13 of whom developed bNAbs. Antibody-dependent cellular phagocytosis (ADCP), complement deposition (ADCD), cellular cytotoxicity (ADCC) and cellular trogocytosis (ADCT) were detected in almost all individuals with levels of activity increasing over time. At 6 months post-infection, individuals with bNAbs had significantly higher levels of ADCD and ADCT that correlated with antibody binding to C1q and FcγRIIa respectively. In addition, antibodies from individuals with bNAbs showed more IgG subclass diversity to multiple HIV antigens which also correlated with Fc polyfunctionality. Germinal center activity represented by CXCL13 levels and expression of activation-induced cytidine deaminase (AID) was found to be associated with neutralization breadth, Fc polyfunctionality and IgG subclass diversity. Overall, multivariate analysis by random forest classification was able to group bNAb individuals with 85% sensitivity and 80% specificity based on the properties of their antibody Fc early in HIV infection. Thus, the Fc effector function profile predicted the development of neutralization breadth in this cohort, suggesting that intrinsic immune factors within the germinal center provide a mechanistic link between the Fc and Fab of HIV-specific antibodies.

## Author summary

Some HIV-infected individuals develop antibodies that are capable of neutralizing the majority of HIV strains, a highly desirable function mediated by the antibody Fab portion. While antibodies elicited by current vaccines have failed to recreate this activity, the partial protection seen in the RV144 vaccine trial has been attributed to antibody Fc-mediated effector functions such as cell killing. In this study, we found that HIV-infected individuals who show a diversified and potent Fc response early in infection were more likely to develop broadly neutralizing antibodies later on. Examination of B cell functions associated with good germinal center activity, provided evidence for a common mechanistic link between the regulation of the Fc and Fab mediated activities in these individuals. Our finding of an Fc effector function profile that arises early and predicts neutralization breadth could be used in the evaluation of vaccine candidates designed to generate neutralizing antibodies. Common immune determinants associated with both Fab and Fc function could furthermore be exploited for vaccine design to harness the full potential of HIV-specific antibodies.

## Introduction

Antibodies form a link between the adaptive and innate immune systems and serve as a correlate of protection for many viral vaccines. They mediate diverse functions through the use of the Fab portion to bind specific antigens and the Fc portion that interacts with cellular receptors to effect a variety of additional non-neutralizing activities [1]. As yet, no HIV vaccine has been able to elicit broadly neutralizing antibodies (bNAbs), however moderate efficacy in the RV144 vaccine trial correlated with Fc-mediated antibody-dependent cellular cytotoxicity (ADCC) in the absence of IgA response generating intense interest in understanding how these responses evolve [2–8]. Maturation of antibodies occurs in the germinal center where they undergo somatic hypermutation to generate high affinity neutralizing antibodies, as well as class-switch recombination to select constant regions that determine the scope of Fc effector functions. Both processes are dependent on the enzyme activation-induced cytidine deaminase (AID) as well as the cytokine milieu within the germinal center, suggesting that the Fab and Fc maturation processes of antigen-specific antibodies may be jointly regulated [9–11].

Although current vaccination strategies are unable to induce bNAbs, approximately 10–30% of individuals produce bNAbs during the course of HIV infection, with only 1–2% classified as elite neutralizers [12–16]. Several factors have been associated with the development of neutralization breadth, including duration of infection, high viral load, low CD4 count, genetic subtype and viral diversity [12, 13, 16–18]. The role of host immunological factors is less clear but includes the maintenance of a high level of T follicular helper cells, increased levels of CXCL13 early in infection, autoimmunity and ethnicity [16, 19–22]. Isolation of bNAbs has revealed that they have unusual features, most notably a high level of somatic hypermutation, which is essential for mediating neutralizing activity [23, 24]. Given the importance of bNAbs for HIV vaccine design, considerable effort has been invested in understanding how these antibodies evolve [25]. However, far less is known about the corresponding Fc effector response and IgG subclass usage in individuals who develop neutralization breadth.

The antibody Fc portion mediates a wide range of functions that are dependent on their affinity for activating and inhibiting Fc receptors, lectins and complement proteins [26]. These functions include phagocytosis of pathogens by macrophages and monocytes, ADCC or direct lysis by natural killer cells, complement deposition and trogocytosis, an Fc dependent exchange of membrane proteins on the surface of an infected to an uninfected cell which may result in cell death [27, 28] (Richardson, et al., submitted). Fc effector functions are modulated at the B cell level by biochemical properties such as glycosylation at a conserved site in the CH2 region and isotype or subclass selection determined by the cytokine milieu in the germinal centers [29–32]. Affinity and specificity for the antigen as determined by the Fab portion has also been shown to impact Fc effector functions [33–35].

Several studies have shown that Fc effector function may influence HIV disease progression, viral control and infant mortality [36–43]. Co-ordination between Fc effector functions has also been associated with HIV control [43, 44], which was also observed in the responses to the partially protective RV144 vaccine [3, 5]. Furthermore, protective immunity in an adenovirus-26 based vaccine in primates was linked to Fc polyfunctionality [45]. However, direct evidence that non-neutralizing antibodies can protect via Fc-mediated functions is limited [46–48] although one study showed a reduced number of transmitted/founder viruses as a result of Fc activity [49]. More recently, non-neutralizing mAbs have been shown to clear HIV-infected cells and protect against infection in humanized mice, despite being less effective than bNAbs [50]. Perhaps more convincing is the accumulating evidence that Fc binding is required for bNAbs to optimally protect from infection, supress viral load or clear infected cells [51–55].

This study aimed to investigate Fc effector functions and kinetics in the context of a broad neutralizing response. Furthermore, we examine potential modulators of this response including binding to Fc receptors, IgG subclass diversity and the interplay between germinal center activity, Fc polyfunctionality and neutralization breadth. Our data show that individuals with bNAbs also have higher Fc polyfunctionality and increased subclass diversity, which was associated with greater AID expression in B cells. This study suggests that the Fc and Fab portions of HIV-specific antibodies are co-ordinately regulated and identifies an Fc effector profile that is associated with the development of bNAbs.

## Results

### HIV-specific Fc effector functions in individuals with and without bNAbs

We aimed to determine whether individuals with HIV-1 specific bNAbs showed differences in Fc effector functions and what the kinetics of these responses were relative to the development of neutralization breadth. Plasma IgG was isolated from 13 bNAb and 10 no-bNAb individuals at 6, 12 and 36 months post-infection matched for viral load (S1 Table and S1A Fig), a major driver of immune activation and neutralization breadth [12, 56]. These were tested in 4 different Fc effector assays measuring ADCC (% granzyme B release), ADCP (% fluorescent bead phagocytosed x MFI of phagocytosed beads), ADCD (% C3b deposition x MFI of deposited C3b) and ADCT (% PKH-26 stained membrane transferred to CFSE+ macrophages). Three different HIV envelope glycoproteins were used to detect HIV-specific Fc responses including gp120 ConC, gp140 C.ZA.1197MB and gp120 CAP45. Transmitted/founder or acute viruses from 10 bNAb and 7 no-bNAb individuals for which sequences were available were used to calculate divergence from the sequence of the three antigens. We found that both groups showed equivalent levels of sequence divergence from each antigen, indicating no bias in terms of antigen binding based on autologous virus sequence (S1B Fig). Furthermore, analysis of longitudinal gp160 sequences in 8 bNAb and 5 no-bNAb individuals with available data showed that viral diversity did not differ between the 2 groups, perhaps not unexpected given they were matched for viral load (S1C Fig).

**Fig 1:**
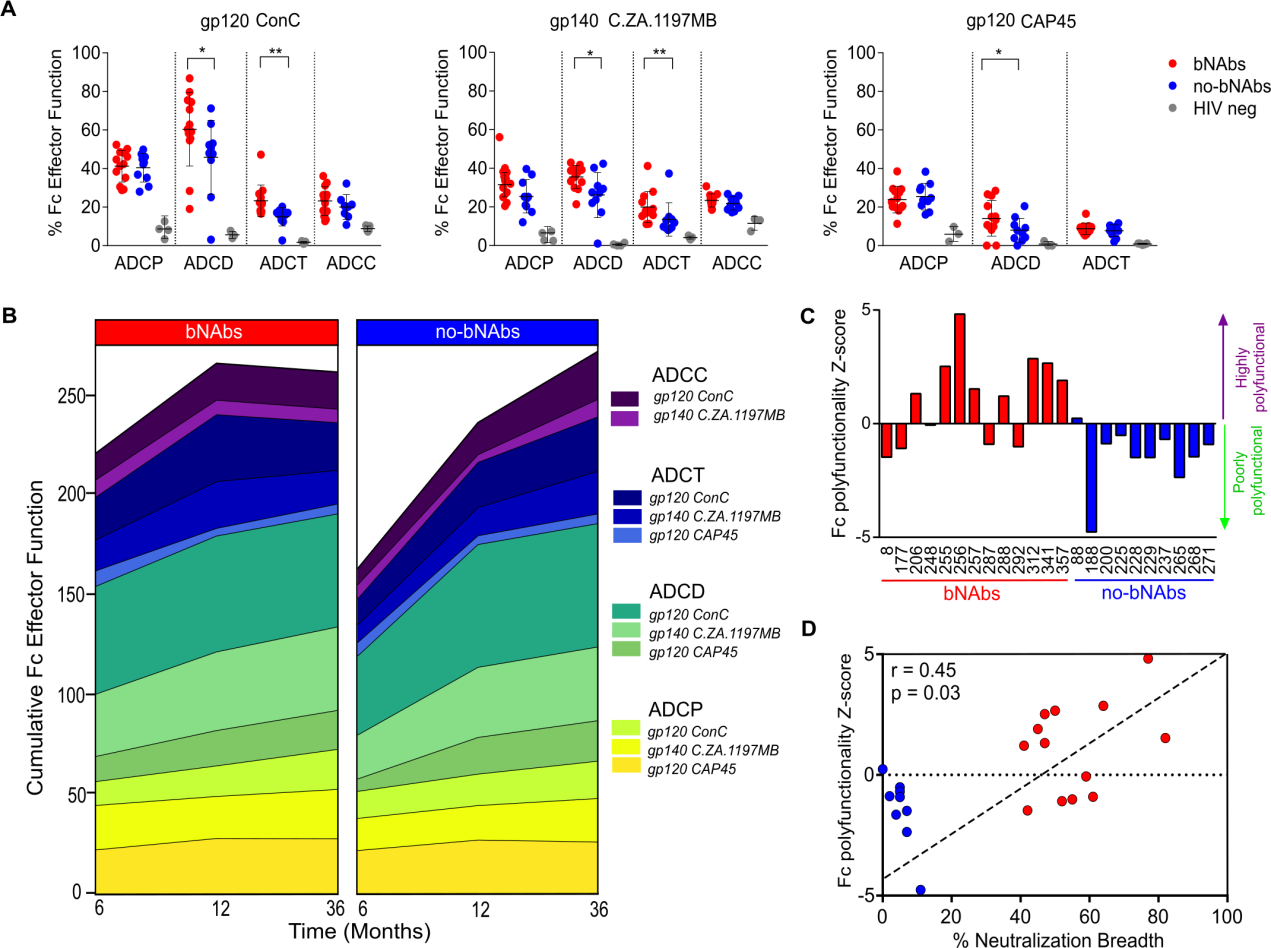
Fc effector function early in HIV infection is higher in individuals that develop bNAbs. (**A**) Purified IgG from 13 bNAb, 10 no-bNAb and 5 HIV-negative individuals (in red, blue and grey respectively) at 6 months post-infection was tested for antibody dependent cellular phagocytosis (ADCP), complement deposition (ADCD), cellular trogocytosis (ADCT) and cellular cytoxicity (ADCC) using three HIV-specific antigens gp120 ConC, gp140 C.ZA.1197MB and gp120 CAP45.G3. Significant differences between groups determined by the Mann-Whitney U test are indicated by *p<0.05; **p<0.001. (**B**) Medians and IQR of different Fc effector functions for bNAb and no-bNAb individuals against all tested antigens over 36 months of infection are indicated as cumulative Fc effector function. Data are representative of 3 independent experiments. (**C**) Each Fc function was standardized by calculating a Z-score and polyfunctionality determined by addition of the Z-scores for all functions for each individual. Bars above the x-axis indicate Fc polyfunctional individuals, while those below indicate poor Fc polyfunctionality. bNAb and no-bNAb individuals are indicated in red and blue respectively. (**D**) Spearman´s correlation coefficient for the relationship between the Fc polyfunctionality Z-score and % neutralization breadth calculated by a 44 multi-clade virus panel is shown. The dashed diagonal line indicates the trend of the relationship.

IgG from almost all individuals, irrespective of the presence of bNAbs, had Fc effector function activity with ADCP and ADCD being the most readily detectable, particularly in response to gp120 ConC (Fig 1A). At 6 months of infection, IgG from bNAb individuals showed significantly higher ADCD against gp120 ConC (p = 0.030), gp140 (p = 0.042) and gp120 CAP45 (p = 0.047) compared to no-bNAb individuals. This was also noted at 12 months for gp140 (p = 0.030) (S2 Fig). ADCT was higher in bNAb individuals at both 6 and 12 months post-infection against gp120 ConC (p = 0.002; p = 0.030) and gp140 (p = 0.004; p = 0.014). There was no difference observed between the 2 groups for ADCC and ADCP against any of the antigens tested but all were significantly higher than the HIV-negative IgG control.

A comparison of the cumulative activity of all 4 Fc effector functions against all 3 antigens over 36 months of infection between the two groups highlighted the differences seen at 6 months (Figs 1B and S2). Significant increases over time were noted primarily in the no-bNAb group for ADCD and ADCC and in the bNAb group for ADCT while high levels of ADCP were maintained in both groups (S2 Fig). No-bNAb individuals had a steeper increase in function over time, as a result of a lower initial Fc effector response. Regardless of neutralization breadth, Fc effector function in both groups was comparable by 36 months. Adsorption of broad neutralizing activity from an individual with bNAbs (CAP255) revealed a significant 3-fold reduction in ADCC, ADCP and 2-fold reduction in ADCD against gp120 ConC compared to the unadsorbed IgG (S3 Fig). This suggests that the N332-specific antibodies that mediate broad neutralizing activity in CAP255 [12], were also responsible for a significant proportion of Fc effector functionality.

### Individuals with bNAbs show a distinct Fc effector polyfunction profile

In order to account for the contributions of all four effector functions equitably, we calculated an Fc polyfunctionality Z-score using the gp120 ConC data from 6 months post-infection. Z-scores with values greater than zero indicated samples with good Fc polyfunctional activity. Of the 13 bNAb individuals, 8 showed positive Z-scores compared to only 1 of the no-bNAb individuals who had a weakly positive Z-score (Fig 1C). The remaining 9 no-bNAb samples had negative Z-scores compared to 5 in the bNAb group. Thus IgG samples from individuals who later went on to develop bNAbs could be distinguished from no-bNAb individuals on the basis of their higher overall polyfunctional polyclonal response against gp120 ConC early in infection. Furthermore, neutralization breadth measured at 3 years post-infection correlated significantly with the Fc polyfunctionality Z-score calculated using data from 6 months of infection (r = 0.45, p = 0.030, Fig 1D). There was also a correlation between these gp120 ConC Z-scores and those calculated using gp140 (r = 0.69, p<0.001, S4A Fig), an antigen that also showed a significant correlation with neutralization breadth (S4B Fig). Fc polyfunctionality Z-scores calculated using CAP45.G3 gp120 did not distinguish bNAb individuals unlike the other 2 antigens (Figs 1C and S4). This is likely to be as a result of CAP45.G3 gp120 having significantly lower titers of binding IgG antibodies than both ConC gp120 and C.ZA1197MB gp140 (S5A Fig). In order to assess collaboration between different Fc effector functions, correlation coefficients between the functions were calculated. There was significant discordance in the no-bNAb group with negative correlations noted between ADCP and all other functions and concordance between ADCT and ADCD (S4C Fig). In contrast, the bNAb group showed no significant discordance or concordance between Fc effector functions, further highlighting that individuals with and without bNAbs have distinct Fc effector function profiles early in HIV infection.

### Antibody binding to Fc receptors and complement is significantly higher in individuals with bNAbs

**Fig 2:**
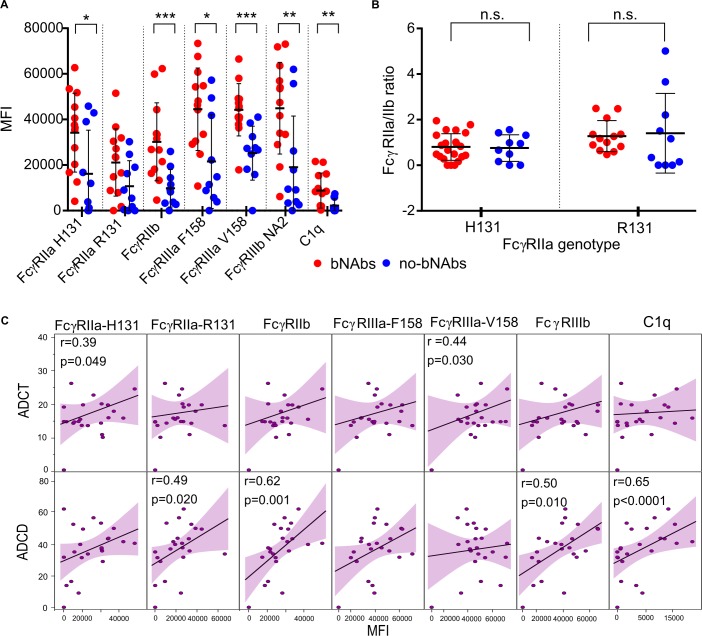
IgG isolated from individuals that develop bNAbs shows increased gp120-specific binding to Fc receptors and complement proteins. (**A**) Binding gp120 ConC-specific IgG isolated from bNAb (red) and no-bNAb (blue) individuals to Fc receptors and C1q measured by an antigen-specific Fc receptor multiplex array. Significant differences (calculated by Mann-Whitney U test) in binding are shown as *p<0.05; **p<0.001; ***p<0.0001. Data are representative of 2 independent experiments. (**B**) The ratio of activating FcγRIIa (either H131 or R131) to inhibitory FcγRIIb receptor binding at 6 months post infection for bNAb and no-bNAb individuals. Medians are shown and significance was calculated by the Mann-Whitney U test. (**C**) Correlations between ADCT or ADCD and binding to Fc receptors and C1q shown as MFI. Significant Spearman´s correlation coefficients are indicated. Lines indicate the trend of the correlations.

Since antibody function is modulated by binding to different Fc receptors and complement proteins, we assessed the binding of gp120 ConC-specific IgG to 6 different Fc receptors and C1q (the first subcomponent of the C1 complex of the classical pathway of complement activation) using a multiplex-based assay (as described in [57]). Polymorphisms of the FcγRIIa (both R131 and H131) and FcγRIIIa (both F158 and V158) receptors that affect IgG binding and impact HIV disease progression (reviewed in [58]) were also tested. Individuals with bNAbs had significantly higher levels of binding to all receptors except FcγRIIa-R131 where only a trend was evident (Fig 2A). Since FcγRIIa is the primary receptor that mediates phagocytosis and this process is negatively regulated by FcγRIIb [59], an increase in binding to both activating and inhibitory receptors among bNAb individuals was somewhat unexpected given that we observed no differences in phagocytosis between the two groups (Fig 1A). We therefore determined the ratio of antibody binding to these 2 receptors which has previously been shown to correlate with phagocytic function [59, 60]. Here we found no differences between the bNAb and no-bNAb groups irrespective of the FcγRIIa-H131/R131 genotype which was more consistent with the finding of similar ADCP levels in the two groups (Fig 2B). ADCT correlated with binding to FcγRIIa and FcγRIIIa (Fig 2C), two receptors that have been implicated in mediating this function [61, 62] (Richardson, et al., submitted). In addition, we found several correlations for ADCD including FcγRIIa-R131, FcγRIIb and FcγRIIIb, however C1q showed the strongest correlation consistent with this being the primary binding protein for complement deposition (r = 0.65; p <0.001).

### Individuals who develop bNAbs have a greater IgG subclass diversity

**Fig 3:**
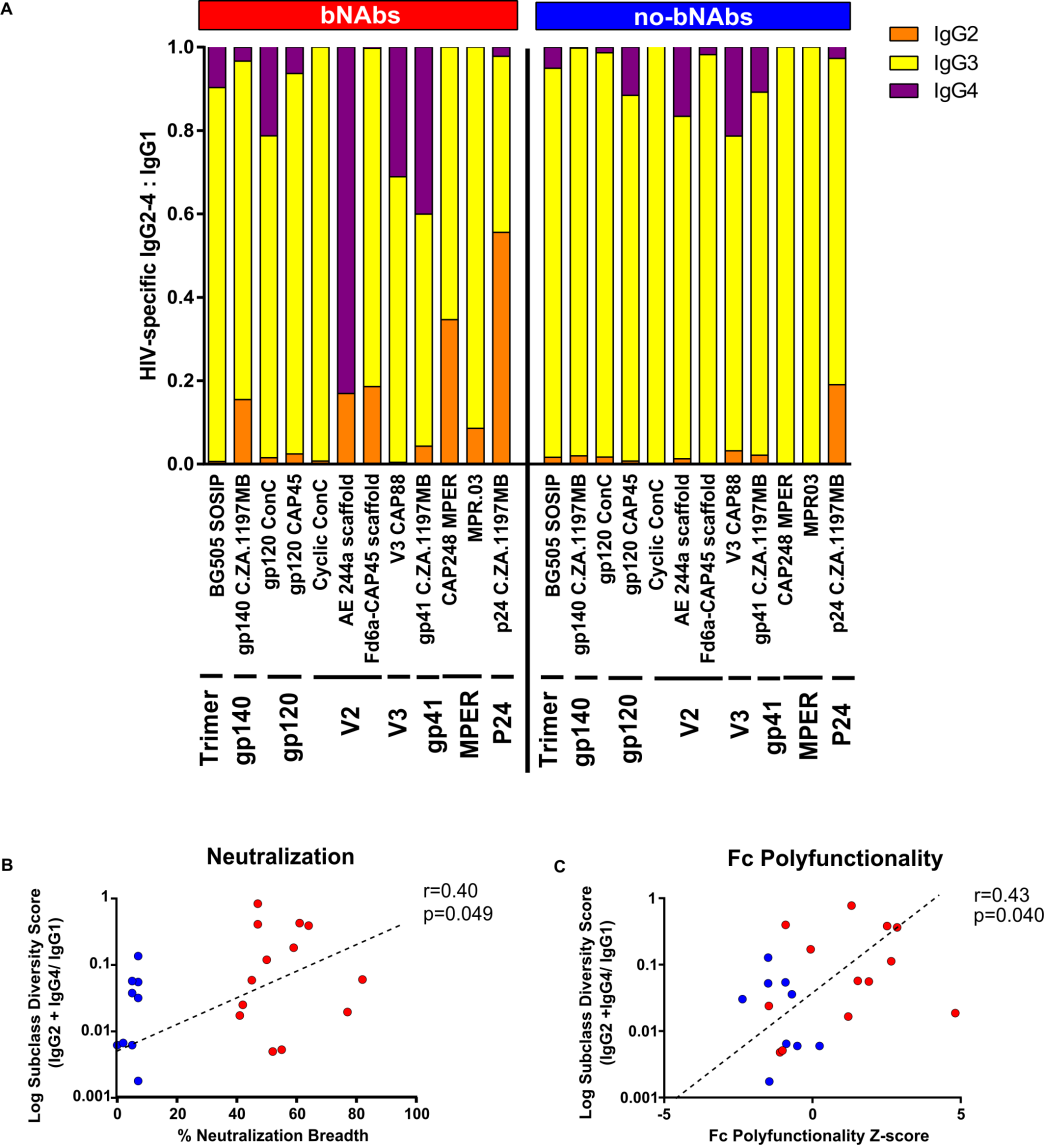
bNAb individuals have higher HIV-specific IgG subclass diversity. (**A**) A multiplex assay was used to measure levels of HIV-specific IgG subclasses present in 6 month samples from bNAb and no-bNAb individuals to 12 different HIV antigens. Median abundance of antigen-specific IgG2, IgG3 and IgG4 (orange, yellow and purple respectively) are represented as a ratio to IgG1 calculated using median fluorescence intensities. Data are representative of 2 independent experiments. Spearman´s correlations between subclass diversity score and (**B**) neutralization breadth and (**C**) Fc polyfunctionality are shown. The score was calculated as the ratio of gp120 ConC IgG2 and IgG4 relative to IgG1 levels. bNAb individuals are shown in red and no-bNAb in blue with dotted trend lines.

IgG comprises 4 subclasses each with distinct Fc regions that bind differentially to cellular Fc receptors and complement proteins. Using an antigen-specific IgG subclass multiplex assay, we measured the levels of IgG1-4 and total IgG against 12 HIV antigens including trimeric envelope, gp140, monomeric gp120, V2, V3, gp41, membrane proximal external region (MPER) and p24. First, to determine if there was any IgG subclass bias of antibodies able to mediate Fc effector functions, we analysed 6-month data with gp120 ConC as this antigen was used in all assays. Other than gp120-specific total IgG that was significantly associated with ADCD (r = 0.74; p<0.001) and ADCC (r = 0.46, p = 0.010), only ADCT was shown to correlate with HIV-specific IgG3 (r = 0.48; p = 0.02) suggesting that multiple rather than single subclasses mediate Fc effector functions (S5B Fig). Our earlier finding that bNAb individuals had greater binding to FcγRIIa-H131 and FcγRIIIa-V158 (Fig 2A), receptors known to bind IgG2 and IgG4 [63] led us to explore whether individuals with bNAbs had a greater diversity of IgG subclasses specific to HIV. While there were no significant differences in HIV-specific IgG1 and IgG3 levels (as a proportion of total antigen-specific IgG) individuals with bNAbs showed significantly higher IgG2 and IgG4 binding to 7 of the 12 antigens tested, including BG505 SOSIP.664 gp140 trimeric protein (S6 Fig). This was also reflected when we represented the less frequent subclasses (IgG2, IgG3 and IgG4) as a proportion of IgG1 (Fig 3A). Overall, we noted that individuals who developed bNAbs showed a greater relative abundance of IgG2 and IgG4 to IgG1. In particular, IgG2 binding to MPER and p24 antigens and IgG4 binding to the A244 V2 antigen, gp120 ConC and gp41 were higher among bNAb individuals. Among the no-bNAb samples, levels of IgG2 and IgG4 were considerably lower with only a V2 antigen and p24 showing some reactivity. We calculated a subclass diversity score based on levels of IgG2 plus IgG4 relative to IgG1 and found this to be correlated with neutralization breadth (r = 0.40, p = 0.049, Fig 3B) and the Fc polyfunctionality Z-score (r = 0.43, p = 0.040, Fig 3C). These data suggested that individuals who develop bNAbs in chronic infection undergo increased IgG class switching within the first 6 months of infection.

### Germinal center activity and AID expression is higher in individuals with bNAbs and associated with Fc polyfunctionality and IgG subclass diversity

**Fig 4:**
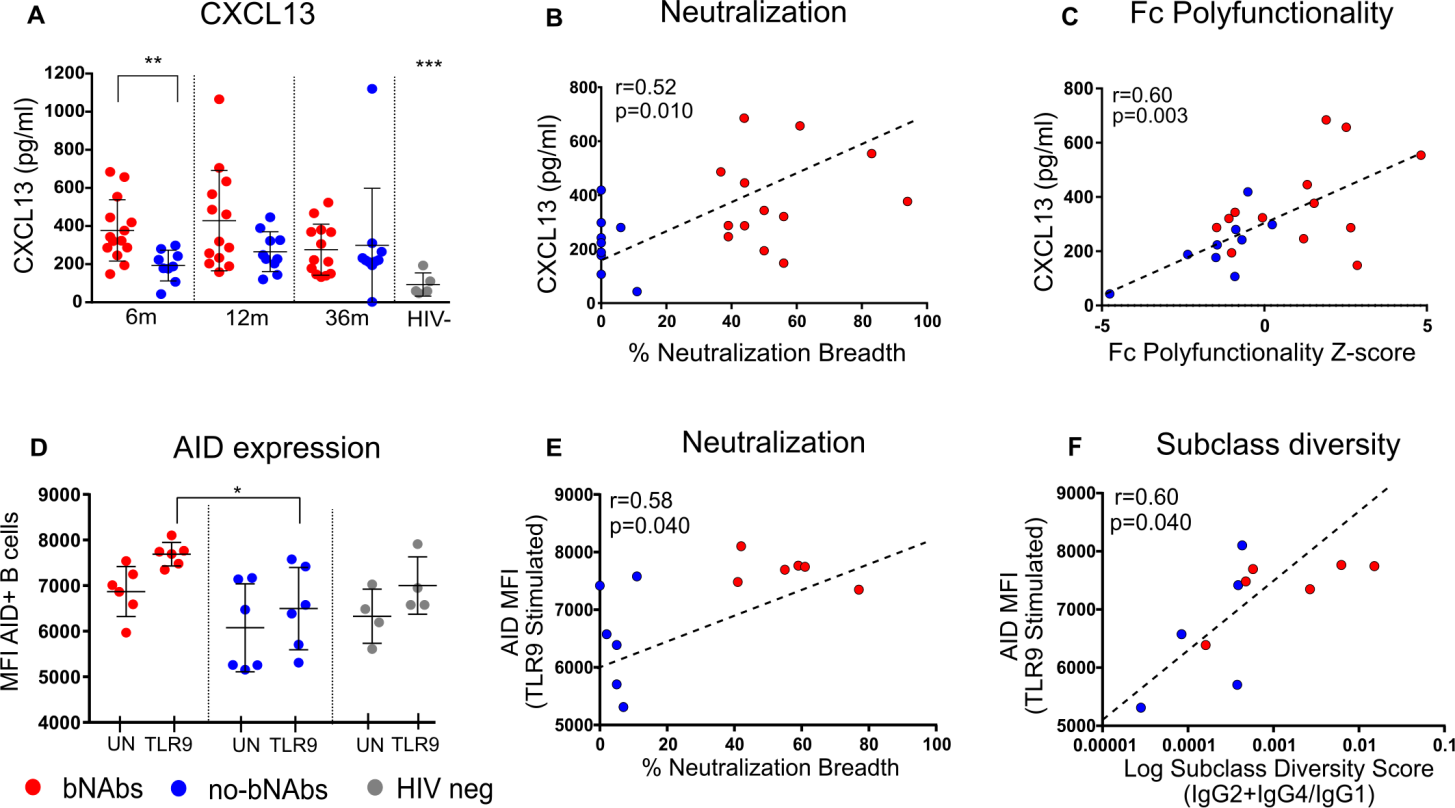
CXCL13 and AID correlate with neutralization breadth, subclass diversity and Fc polyfunctionality. (**A**) Germinal center activity as represented by CXCL13 levels were measured by ELISA in plasma (pg/ml) at 6, 12 and 36 months post infection from 13 bNAb (red), 10 no-bNAb (blue) and 4 HIV-negative individuals (grey). Significance was defined by the Kruskal-Wallis test with Tukey correction where **p = 0.002 and ***p<0.0001. Median values are shown and results are representative of 3 independent experiments. Significant Spearman´s correlations at 6 months post-infection between CXCL13 and (**B**) neutralization breadth and (**C**) Fc polyfunctionality are shown. (**D**) AID was measured in B cells in peripheral blood from 6 bNAb (red), 6 no-bNAb (blue) and 4 HIV-negative (grey) individuals by flow cytometry after stimulation and activation with TLR9 for 3 days. AID expression in live B cells is represented by AID MFI and significance is indicated as *p<0.05, Kruskal-Wallis test with Tukey correction in TLR9 stimulated B cells. (UN = unstimulated; TLR9 = TLR9 stimulated). Significant Spearman´s correlations between AID expression and (**E**) neutralization breadth and (**F**) subclass diversity (calculated by gp120 ConC specific IgG2+IgG4/IgG1) are shown. Dotted trend lines are indicated.

Both class switch recombination and somatic hypermutation take place in germinal centers and are enabled by activation-induced cytidine deaminase (or AID) as well as interactions between T helper follicular (Tfh) cells with B cells. The cytokine CXCL13, expressed by Tfh cells [64, 65], has been shown to correlate with neutralization breadth early in HIV infection and is defined as a marker of germinal center activity [19–21, 56, 66]. We measured CXCL13 levels in plasma by enzyme linked immunosorbent assay (ELISA) and found significantly higher levels in individuals with bNAbs at 6 months post infection (p = 0.002) but not at later time points (Fig 4A). HIV-negative samples were significantly lower than HIV-positive plasma (p<0.001) similar to other studies showing that HIV infection is associated with increased expression of this cytokine [21]. The levels of CXCL13 at 6 months were also correlated with neutralization breadth (r = 0.52, p = 0.010, Fig 4B) and Fc polyfunction measured against gp120 ConC (r = 0.60, p = 0.003, Fig 4C). CXCL13 was not correlated with IgG subclass diversity or total IgG levels.

We next directly measured AID in B cells from 6 bNAb individuals and 6 no-bNAb individuals from 6 months post infection, from whom PBMC were available. In order to detect AID at a reliable level we stimulated PBMCs for 3 days with TLR9 and confirmed stimulation by the co-staining of live B cells (defined as CD3/CD16/CD14- CD19+ as in S7A Fig) with AID and with Ki67 (a marker of proliferating cells). As expected, stimulation increased the percentage of B cells expressing AID but no significant difference was observed among the 2 groups or HIV-negative individuals (S7B Fig). However, when AID expression was measured by the mean fluorescence intensity (MFI) of AID, we noted significantly higher expression of AID in stimulated B cells from individuals with bNAbs compared to no-bNAbs and HIV-negative individuals (p = 0.010, Kruskal-Wallis test with Tukey correction for multiple comparisons, Fig 4D). Even on unstimulated B cells, median AID expression levels tended to be higher in bNAb individuals. Interestingly, both neutralization breadth (r = 0.58; p = 0.040, Fig 4E) and IgG subclass diversity (r = 0.60, p = 0.040, Fig 4F) correlated with AID expression suggesting germinal center activity not only plays a role in the development of bNAbs but is also an indicator of enhanced Fc effector function.

### Random forest classification defines bNAb individuals based on Fc effector functions

**Fig 5:**
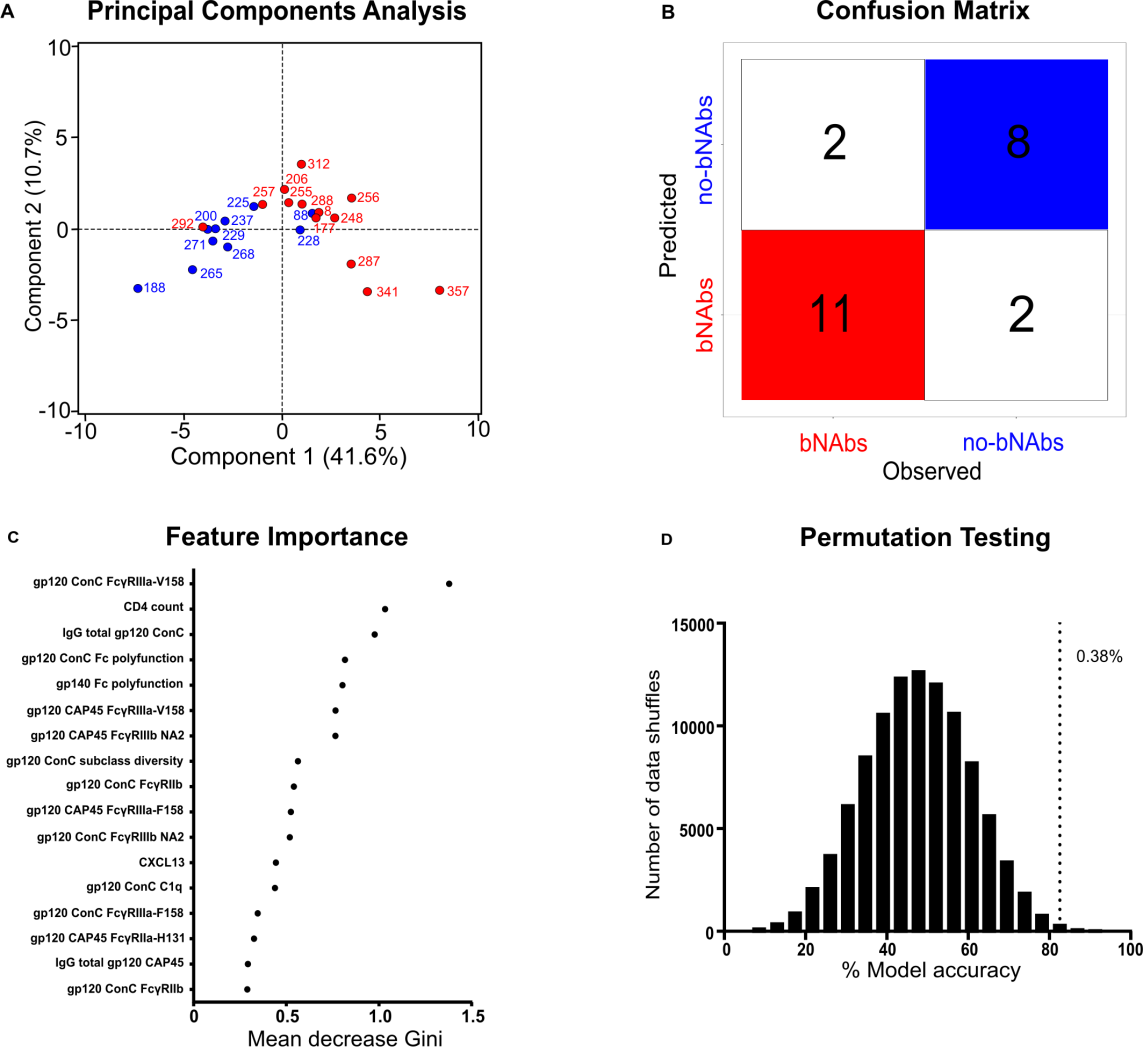
Multivariate classifications reveal that individuals who develop bNAbs can be reliably identified by their Fc features at 6 months of infection. (**A**) Principal components analysis of 13 bNAb (red) and 10 no-bNAb (blue) using 17 variables. Individual CAPRISA identifiers are shown, with component 1 and 2 explaining 52.3% of the variance in the data set. (**B**) Confusion matrix showing the classification of bNAb and no-bNAb individuals achieved by random forest classification. Shown are the numbers of individuals for each predicted or observed group with correct classifications indicated in color and misclassifications indicated in white. The 2 bNAb (CAP257 and CAP292) and 2 no-bNAb (CAP88 and CAP228) individuals that were incorrectly classified can be seen in 5A. (**C**) Importance of the features employed in the random forest classification is indicated by the mean decrease in Gini importance weighting. (**D**) The model was verified by permutation testing following random shuffling of the classification data 100,000 times. The dashed line indicates the accuracy of the proposed model (82.6%), with shuffles resulting in accuracy greater than this shown as a proportion of the total shuffles (0.38%).

In order to ascertain which of the 29 variables examined in this study were best able to separate bNAb from no-bNAb individuals, we used Spearman’s correlations that were adjusted for multiple comparisons by the Benjamini–Hochberg method (S2 Table). Owing to the small sample size of the individuals tested for AID, this variable was omitted. Subclass diversity, Fc receptor and C1q binding antibodies targeting gp120 from both CAP45 and ConC, Fc polyfunctionality and CXCL13 were all significantly associated with the bNAb group. CD4 count was significantly negatively associated with the bNAb group and, as expected, we saw no association with viral load due to matching of the two groups for this variable. The 17 variables that showed significance, were used in further statistical analyses to refine groupings. When subjected to a principal components analysis, the sum of the first 2 components were able to explain 52.3% of the variability of the data set (Fig 5A). Furthermore, when random forest classification was used to define bNAb and no-bNAb groups, it did so with 85% sensitivity 80% specificity, and 82.6% accuracy, only misidentifying 2 bNAb and 2 no-bNAb individuals shown in the confusion matrix in Fig 5B. The features important for this classification are represented by the Gini importance weighting, and identified increased binding to gp120 ConC FcγRIIIa-V158, low CD4 count, gp120 ConC-specific total IgG and Fc polyfunction for gp120 ConC and gp140 as being the best features to classify bNAb individuals (Fig 5C). We then tested the strength of the model by permutation testing, allowing for 100,000 random data shuffles, revealing that there was a 0.38% probability that the same model could be obtained at random (Fig 5D). Collectively, these analyses indicate that Fc properties can be used to reliably discriminate between individuals who develop bNAbs from those who do not.

## Discussion

In this study, we found that HIV-infected individuals who develop bNAbs also have a distinct Fc effector function profile and increased subclass diversity associated with markers of enhanced germinal center activity. Specifically, these individuals showed more potent trogocytosis and complement deposition as well as IgG2 and IgG4 responses to multiple HIV antigens early in infection compared to individuals who did not develop bNAbs. Interestingly, both CXCL13 levels in plasma and AID expression levels in activated B cells correlated with neutralization breadth and Fc polyfunctionality. Our data suggest that markers of germinal center activity that link Fab and Fc could be exploited for vaccine design to harness the full potential of HIV-specific antibodies.

To conduct this study we made use of longitudinal samples collected from HIV-1 infected participants in the CAPRISA acute infection cohort in which the development of bNAbs has been well described [12]. We selected all 13 participants who developed bNAbs by 3 years of infection as well as 10 matched no-bNAb controls. Less than 20% of individuals in the CAPRISA cohort developed bNAbs, similar to many other studies, and so we selected all 13 available bNAb individuals and matched them to those with no-bNAbs that had similar viral levels. While this is not representative of the general population, our aim was to compare those 2 distinct phenotypes in order to maximize our ability to detect differences associated with bNAb development. We found that, unlike neutralization, Fc effector function was detected early in infection in the majority of individuals. Furthermore, levels of ADCD, ADCT, ADCC increased over time while ADCP remained high throughout 3 years of infection. Other studies that have examined the kinetics of ADCC [36, 67–69] and ADCP [70–72] from acute to chronic HIV infection have shown inconsistent results with some showing higher activity early in infection while others suggest, similar to our study, that these activities increase over time. These differences are likely due to the use of a variety of assays and antigens and supports the efforts being made to standardise Fc effector assays [43]. Interestingly, we found differences in Fc effector functions between bNAb and no-bNAb individuals at 6 months post-infection prior to the development of neutralization breadth. Other factors previously shown to be associated with neutralization breadth, such as high viral load and low CD4 count, in this and other cohorts, were also seen early in infection [12, 14, 18, 20]. However, at 3 years post-infection we found no differences in Fc effector function between the two groups further highlighting that events early in HIV infection are signatures, but not necessarily drivers, for the later development of neutralization breadth.

Positive correlations between different Fc effector functions have previously been reported among elite controllers and vaccinees in the RV144 trial suggesting a co-ordinated immune response in these groups [5, 44]. Our study showed that while bNAb individuals did not show significant concordance between Fc functions there was also no significant discordance between the various functions. This differentiated them from no-bNAb individuals who showed significant discordance, which has also been seen in chronic HIV infection [43]. Only one other study has examined Fc effector function in the context of neutralization breadth and found that ADCC did not differ between the groups, similar to our findings [69]. Here we extend this to examine additional Fc effector functions and find that bNAb individuals could be distinguished from no-bNAb individuals by having higher levels of ADCD and ADCT at 6 months of infection. Recent data suggested that ADCD may have been a correlate of reduced risk in RV144 as a result of V2-specific antibodies efficiently activating complement [73]. In our study, ADCD was strongly correlated with total HIV-specific IgG levels which may have facilitated increased IgG complex formation in individuals that develop bNAbs [74]. Furthermore, binding of C3 components to CR2 (complement receptor 2) on the surface of follicular dendritic cells (FDC) in the germinal center facilitates the presentation of antigen to B cells during the processes of affinity maturation and isotype switching [75, 76]. Thus our finding of higher levels of C3b deposition by IgG present in bNAb individuals could contribute to increased antigen presentation through binding to CR2 on FDCs. In contrast, increased ADCT was not due to higher IgG levels although it was the only Fc effector function that showed a correlation with a single IgG subclass, namely IgG3. Since IgG3 preferentially binds to higher affinity polymorphic variants FcγRIIa-H131 and FcγRIIIa-V158 this may explain the associations we observed between binding to these receptors and ADCT. While the role of Fc-mediated trogocytosis in HIV infection has not yet been explored, one potential mechanism that has been reported in studies of cancer is that repeated “nibbling” of membrane proteins on tumorigenic cells by effector cells can result in cell killing [27]. Others have shown ADCT facilitates the deposition of “snatched” antigen-specific IgG on the surface of effector cells, potentially increasing antigen presentation for T cell help [77–79]. Whether either of these mechanisms contributed to the development of neutralization breadth will require an analysis of the antigens captured by ADCT and if these are able to enhance HIV-specific immune responses when deposited on antigen-naïve cells.

The relative abundance of IgG2 and IgG4 was higher in bNAb individuals across multiple HIV antigens and this correlated with neutralization breadth. In support of this, we found that antibodies from bNAb individuals had higher binding to FcγRIIa-H131 and FcγRIIIa-V158 as compared to the respective lower affinity polymorphic variants R131 and F158. H131 and V158 are known to bind more efficiently to IgG2 and IgG4 respectively although these levels are still significantly lower than IgG1 and IgG3 [63, 80]. This may reflect increased relative abundance of these subclasses in bNAb individuals which may also explain why we saw no differences in ADCC. Both IgG2 and IgG4, located downstream of IgG3 and IgG1 on chromosome 14 [81, 82], have been shown to dampen down the inflammatory response by competing with IgG1 [83, 84]. Furthermore, IgG4 binds FcγRIIb, the only inhibitory Fc receptor, with a higher affinity than all other subclasses [80]. Thus the use of multiple IgG subclasses in bNAb individuals may help to balance the highly pro-inflammatory activities of IgG1 and IgG3 early in infection, increasing the diversity of the antibody response [85] and potentially promoting events that are required for later development of neutralization breadth. Although HIV-specific IgG2 and IgG4 showed greater relative abundance, it should be noted that both were still present at very low levels compared to IgG1 and IgG3. Despite this and the fact that IgG2 and IgG4 have reduced Fc effector functionality, subclass diversity in this study still correlated with overall Fc polyfunction. An excess of IgG2 and IgG4 is thought to have compromised the effectiveness of gp120 vaccines possibly due to competition with IgG1 and IgG3 [5, 86, 87]. Thus, in current vaccination strategies we may lack the balance of subclass diversity required to support bNAb development. Indeed, others have found higher isotype diversity, specifically IgG2 levels, among viremic controllers suggesting that a balance of IgG subclasses is a key factor in immune function [82, 85, 88]. Alternatively, subclass diversity may be a secondary consequence of elevated AID activity in individuals with bNAbs. If so, then somatic hypermutation required for bNAb development may come at the expense of IgG1/3 dominance because of increased downstream class switching. Irrespective of the cause, subclass diversity is an important consideration for pathogenic outcomes and may have implications in vaccination strategies [82].

Similar to others, we found that levels of CXCL13 were higher in bNAb individuals early in infection suggesting that this cytokine provides important signals for bNAb development [20, 21, 56, 66]. However, we are the first to examine the relationship between CXCL13 and Fc effector function and find that it correlated more strongly with Fc effector function than with neutralization breadth. Since CXCL13 is necessary for migration of B cells to germinal centers where clonal selection, somatic hypermutation and class-switching occurs, these data suggest that the Fc region of bNAbs are subjected to similar processes. AID plays a more direct role in facilitating somatic hypermutation and class switching explaining the correlation we observed with neutralization breadth as well as subclass diversity of the Fc. Cohen and colleagues have previously shown that AID transcripts were higher in bNAb individuals by *ex vivo* transcriptional profiling [20]. Here we show using the different approach of flow cytometry, that expression of the AID enzyme in activated B cells from bNAb individuals is higher than no-bNAb individuals. Collectively, these data indicate that immune factors associated with germinal centers drive diversification of both Fab and Fc functions.

The Fab and Fc regions are encoded by different genes and class switch recombination produces new effector function in the context of an existing antibody specificity suggesting independent evolution of these two portions. However, several groups have shown that the isotype or subclass of the constant region can subtly affect Fab function such as neutralization and avidity across various diseases [89–92]. In addition, class-switch recombination and somatic hypermutation both require AID to target transcription and DNA cleavage respectively [10]. Our study provides evidence for similar modulation of the Fc and the Fab driven by AID in the context of an HIV-specific polyclonal response. In our model, we show that HIV-specific binding to Fc receptors, Fc polyfunctionality and total HIV-specific IgG levels were among the top five features that classify bNAb individuals. Among these, low CD4 count and high HIV-specific IgG levels have been previously associated with the development of neutralization breadth [12, 13]. Our data support findings from other cohorts showing correlations between neutralization breadth with immunologic analytes, such as CXCL13 [21, 66]. However, we are the first to describe that enhanced complement deposition and trogocytic activity are associated with the development of neutralizing antibodies, which will need to be confirmed in other cohorts. Furthermore, we showed, through adsorption experiments, that bNAbs in one individual were also responsible for Fc effector functions. Whether these functions are mediated by the same or separate antibody molecules will require further study, preferably via the isolation of HIV-specific monoclonal antibodies with their native Fc regions. Nevertheless, our finding that the Fab and Fc functions may be jointly regulated in individuals with bNAbs could be important in the context of vaccination where the aim is to induce a polyclonal response with both neutralizing and Fc effector functionality.

Overall, this study illustrates that the functions and characteristics of the antibody Fc were able to reliably classify bNAb individuals. Furthermore, early Fc function and subclass diversity predicted which HIV-infected individuals went on to develop bNAbs. Unlike broad neutralization, Fc-mediated activities were present in all individuals and suggests that favourable Fc effector function could be elicited as a prelude to a bNAb response which is encouraging for vaccination strategies. Additional studies are required to understand how neutralization and Fc effector functions can be favourably tuned to produce a protective polyclonal response. Our data suggests that common immune factors underlie the development of both neutralization breadth and Fc effector function. Moreover, as Fc effector function differences occur well before the development of neutralization breadth, these properties could be used to identify individuals with the necessary germinal center activity to respond to a vaccine aimed at the generation of bNAbs.

## Materials and methods

### Ethics statement

CAPRISA 002 and 004 acute HIV infection cohorts were approved by the Biomedical Research Ethics Committee of the University of KwaZulu-Natal (M160791) and this specific study was approved by the Human Research Ethics Committee of the University of the Witwatersrand (M150313). All participants were adults and provided written informed consent to have their stored samples used for future studies. All healthy subjects in this study were adults and provided written informed consent to obtain both PBMCs (peripheral blood mononuclear cells) and plasma and have their samples stored for future use.

### Sample preparation and definition of neutralizing breadth

Serum from participants in the CAPRISA 002 and 004 acute HIV infection cohorts [93, 94] were previously screened for neutralization breadth using a 44 virus multi-clade panel [12]. Samples from individuals able to neutralize at least 40% of a 44 virus panel at 3 years post-infection were defined as having developed bNAbs. Individuals with no-bNAbs were also matched based on viral load at 6 months and were not significantly different at all time points tested (S1A Fig). By these criteria, we included plasma samples from 13 bNAb and 10 no-bNAb individuals at 6 months, 1 year and 3 years post-infection (S1 Table). IgG was isolated from plasma using Protein G according to the manufacturer’s instructions in order to eliminate the cofounding impact of cytokines or plasma proteins on cell-based assays, quantified by a nanodrop spectrophotometer (Pierce Biotechnology, Rockford, IL) and confirmed by IgG ELISA. Pooled IgG from HIV-positive samples from the NIH AIDS Reagent programme (HIVIG) was used in all assays to normalise for plate to plate variation while samples from 5 HIV-negative individuals from the same cohort were used as negative controls.

### Proteins and peptides

Plasmids encoding histidine-tagged recombinant gp120 from ConC and gp120 from CAP45.G3 envelope sequences were transfected using polyethylenimine 25 kDa (Polysciences Inc, Warrington, PA) into HEK293T cells obtained from Dr George Shaw (University of Alabama, Birmingham, AL). Cells were cultured at 37°C, 5% CO2 in DMEM containing 10% heat-inactivated fetal bovine serum (Gibco, Gaithersburg, MD) with 50 μg/ml gentamicin (Sigma-Aldrich, St Louis, MO) and disrupted at confluency by treatment with 0.25% trypsin in 1 mM EDTA (Sigma-Aldrich, St Louis, MO). Recombinant proteins were expressed and purified as previously described [95]. BG505 SOSIP.664 gp140 trimer was produced in HEK293F suspension cells (Invitrogen) and purified by size exclusion chromatography (SEC) [96]. Prior to use trimer was subject to quality control by ELISA binding of CAP256-VRC26.25 and PGT151 and the lack of binding to F105. The C.ZA.1197MB strains of gp41, p24 and gp140 was purchased from Immune Tech (Lexington, New York), CAP88. B5 V3 peptide, CAP248 MPER peptide and MPR.03 were purchased from Peptide 2.0 (Chantilly, Virginia). V1V2 scaffolded proteins were expressed in HEK293S cells (ATCC CRL-3022 N-acetylglucosaminyltransferase I deleted), grown in a shaking incubator at 37°C, 5% CO_2_, 70% humidity at 125rpm. Cultures were harvested after seven days and purified by sequential Ni-NTA and SEC.

### Viral divergence and diversity analysis

Measurement of divergence of autologous viral sequences from antigens was done using available transmitted/founder or acute gp120 viral sequences from 10/13 bNAb and 7/10 no-bNAb individuals which were aligned using MUSCLE (MUltiple Sequence Comparison by Log- Expectation https://www.ebi.ac.uk/Tools/msa/muscle/). A script adapted from SONAR (Software for the Ontogenic aNalysis of Antibody Repertoires) [97] was adapted to calculate the percentage nucleotide differences of autologous viruses compared to the antigen sequences (ConC gp120, C.ZA.1197MB gp140 and CAP45.G3 gp120).

Diversity in gp160 was estimated using longitudinal sequences from 8 bNAb and 5 no-bNAb individuals. This included sequences from 6 (between 5 and 28 per individual), 12 (between 8 and 30 per individual) and 36 months (between 5 and 19 per individual) post-infection. CAP256 was superinfected and sequences from both the primary and superinfecting viruses were included. Mean pairwise genetic distances between sequences were calculated using MEGA v7 [98].

### Antibody dependent cellular phagocytosis assay (ADCP)

The THP-1 phagocytosis assay was performed as previously described [99] using 1μM neutravidin beads (Molecular Probes Inc, Eugene, OR) coated with gp120 ConC, gp120 CAP45.G3 or gp140 C.ZA.1197MB. Polyclonal IgG samples were titrated and tested at a final concentration of 100μg/ml. Phagocytic scores were calculated as the geometric mean fluorescent intensity (MFI) of the beads multiplied by the percentage bead uptake. This, including all other flow cytometry work was completed on a FACSAria II (BD biosciences, Franklin Lakes, New Jersey). THP-1 cells were obtained from the NIH AIDS Reagent Program and cultured at 37°C, 5% CO2 in RPMI containing 10% heat-inactivated fetal bovine serum (Gibco, Gaithersburg, MD) with 1% Penicillin Streptomycin (Gibco, Gaithersburg, MD) and not allowed to exceed 4 x 10^5^ cells/ml.

### Antibody dependent cellular cytotoxicity (ADCC)

ADCC activity was detected by the previously described ADCC-GranToxiLux (GTL) assay using antigen-coated cells [100]. This assay was chosen as it is high-throughput and has been previously validated. Whole PBMCs from a healthy donor were used as effector cells. The FcγRIIIa receptor was genotyped as being homozygous for valine at position 158 by the TaqMan SNP genotyping assay (rs396991) (Applied Biosystems, Foster City, CA) to ensure high levels of lysis. Target CEM-NKR.CCR5 (CEM-natural killer resistant T lymphoblast cell line transduced with CCR5) cells were coated with gp120 ConC and gp140 C.ZA.1197MB at 2.5μg/ml and 10μg/ml respectively. Optimal coating concentration was determined by titration of the antigen and measuring residual levels of unbound CD4 with anti-CD4 FITC (SK3 clone, BD Biosciences). MAb A32 was used as a positive control with Palivizumab (MedImmune, LLC; Gaithersburg, MD) used as negative control. The results, analysed in FlowJo (FlowJo LLC, Ashland, Oregon) are expressed as % Granzyme B (GzB) activity, defined as the percentage of cells positive for proteolytically active GzB out of the total viable target cell population. The final results are expressed after subtracting the background represented by the % GzB activity observed in wells containing effector and target cell populations in the absence of IgG. CEM-NKR.CCR5 cells were obtained for the NIH AIDS Reagent programme and were cultured at 37°C, 5% CO2 in RPMI containing 10% heat-inactivated fetal bovine serum (Gibco, Gaithersburg, MD) with 1% Penicillin Streptomycin (Gibco, Gaithersburg, MD).

### Antibody dependent complement deposition (ADCD)

ADCD was determined by the deposition of the complement component C3b on the surface of CEM-NKR.CCR5 cells [3]. Target cells were pulsed with 6μg gp120 ConC, 14μg gp120 CAP45.G3 or 6μg gp140 C.ZA.1197MB in 100μl of R10 media (10% FBS 1% Pen/Strep RPMI, Gibco, Gaithersburg, MD) determined by titration as described above for 1 hour at room temperature and incubated with 100μg/ml of IgG preparation. HIV-negative plasma was used as a source of complement and diluted 1 in 10 in 0.1% gelatin/ veronal buffer (Sigma-Aldrich, St Louis, MO) and 150μl added and incubated for 20 minutes at 37°C. The cells were then washed in 15mM EDTA in PBS and C3b was detected by flow cytometry using an anti-human/mouse complement component C3/C3b/iC3b mAb (Cedarlane, Burlington, Canada). Unpulsed cells were used as background controls and HIV-negative plasma was heat-inactivated at 56°C to remove complement as a negative control. The ADCD score was defined as geometric MFI multiplied by % cells positive for C3b deposition.

### Antibody dependent cellular trogocytosis (ADCT)

CEM-NKR.CCR5 cells were pulsed with gp120 ConC (2.5μg/ml), gp120 CAP45.G3 (25μg/ml) or gp140 C.ZA.1197MB (10μg/ml) for 75 minutes at room temperature. Optimal coating concentrations were determined as described above. Cells were stained with PKH26 dye (Paul Karl Horan 26 dye) as per instructions from the manufacturer (Sigma-Aldrich, St Louis, MO) and resuspended at 2 million cells/ml. IgG at a final concentration of 100μg/ml were added to the cells and incubated for 30 minutes at 37°C. THP-1 cells were stained with intracellular CFSE (carboxyfluorescein succinimidyl ester) and 150μl at 6.7 x 10^5^ cells/ml was added to the plate and incubated for a further hour at 37°C. Cells were then washed with 15mM EDTA in PBS. Flow cytometry was used to distinguish PKH26+ CFSE+ THP-1 cells (i.e. the uninfected monocytes that have received membrane fragments from the coated cells) and are represented as a proportion of total THP-1 cells. Doublets were excluded from the analysis by singlet gating (Richardson, et al., submitted). The assay was gated on stained CEM and THP-1 cells incubated in the absence of IgG to ensure that we did not measure antibody-independent trogocytosis. Uncoated PKH26 stained CEM cells were also incubated with THP-1 cells in the presence of HIV-specific IgG in order to ensure that the responses seen were HIV-specific. HIV-negative IgG as well as Palivizumab were used as negative controls while HIVIG was used to standardise between runs.

### Adsorption of neutralizing activity

Adsorption of broadly neutralizing antibodies that target the N332 glycan from isolated plasma IgG was performed as previously described [101]. For this we used ST09 (1gut-mV3 scaffold), a V3 scaffold that specifically binds N332-directed bNAbs [102] (a gift from Dr Peter Kwong, Vaccine Research Center, NIH, USA) covalently coupled to tosyl-activated magnetic beads. Following adsorption, the depletion of anti-ST09 (1gut-mV3 scaffold) activity was measured by ELISA and reduction of neutralization was confirmed by neutralization assay as described elsewhere [103].

### Customised multiplex IgG subclass assay and Fc binding array

A customised multiplex assay was used as previously described [104]. Briefly, Multiplex microplex carboxylated beads (Luminex, Madison, WI) were coupled to 12 HIV specific antigens. Fifty μl of a 100 microspheres/μl bead preparation was incubated with purified IgG overnight (100μg/μl) at 4°C. Levels of bulk IgG and IgG1-IgG4 were detected by PE-conjugated detection agents (Southern Biotech, Birmingham, AL) by a Bio-Plex200. The mean of PBS only samples added to 3 times the standard deviation was subtracted from all samples.

Similarly, for the Fc binding array, HIV-specific antigen coated microspheres (10 beads/μl) were added to 5μg/ml of purified IgG in black clear bottom 384-well black plates in replicate and incubated for 2 hours. Antigen binding to FcγRIIa (H131/R131), FcγRIIb, FcγRIIIa (F158/V158), FcγRIIIb (NA2) and C1q was detected by incubating the tetrameric PE-conjugated reagents (as described elsewhere [57]) for an hour. HIVIG was used as a positive control and to track plate-to-plate variation.

### CXCL13 ELISA

CXCL13 was detected in plasma using the Human CXCL13 Quantikine ELISA Kit (R&D Systems) as described by the manufacturer.

### Activation-induced cytidine deaminase (AID) detection

PBMCs from selected individuals were thawed in the presence of Benzonase Nuclease (Novagen, Madison, WI) and rested overnight at 37°C. Half a million cells in 200μl of RPMI-10 media was stimulated with 0.5uM TLR9 agonist ODN-2006 (Invivogen, San Diego, CA) for 3 days at 37°C. A total of 1–3 million cells for both the stimulated and unstimulated controls were pooled after 3 days for flow cytometric staining. Surface staining was performed in PBS/0.1% BSA including CD16, CD14, CD3 (APC-CY7), CD19 (PE-CY7), IgD (FITC), CD38 (PE-CY5) (BD Pharmingen, Franklin Lakes, NJ) and was followed by intracellular staining using the BD Cytofix/Cytoperm kit as per the instructions from the manufacturer for AID (AF647, BD) and Ki67 (PerCP.Cy5, eBiosciences, San Diego, CA). B cells were defined as lymphocytes, single cells, live cells (LIVE/DEAD Fixable Dead Cell Stain), CD3-/CD16-/CD14- (APC-CY7), CD19+ (PE-Cy7). A HIV-negative control donor PBMC sample was run in each set of experiments to ensure consistency between runs.

### Statistical analysis

Fc polyfunctionality Z-scores were calculated by standardising each Fc effector function (where the mean of the function is subtracted from the individual value and divided by the standard deviation of the mean) and then adding all the Z-scores for each function per individual. All comparisons between groups were done with non-parametric tests including Mann-Whitney U tests (for two groups) and Kruskal-Wallis tests with Tukey’s correction for multiple comparison and all confidence intervals were at 95%. All correlations reported are non-parametric Spearman’s correlations and all statistical analysis was done with two-sided testing with an alpha level of 0.05. Univariate analysis was performed using GraphPad 6 (GraphPad Software, Inc, La Jolla, CA). For the multivariate analysis, the mean added to 3 standard deviations of the mean of HIV-negative samples for HIV-specific experiments was subtracted from HIV-positive data at 6 months post-infection and was then standardised by Z-score prior to analysis. In addition, P-values of Spearman’s correlations with bNAb and no-bNAb groups was corrected for multiple comparisons by adjusting alpha levels using the false discovery rate (where the FDR was 5%) and Benjamini–Hochberg procedure [105]. Significant features were used in a principal components analysis (PCA) using JMP13 from SAS (Cary, NC). No missing data was imputed. Significant features only were again used to classify individuals into groups in order to decrease overfitting and was done by random forest classification using the R package ‘randomForest’ with the number of trees set at 500 and cross-validation indicated by out-of-bag estimate. Feature weighting or importance in the classification was represented by the mean decrease of Gini importance which measures the average gain of purity by splits of a given variable. If the variable is useful it tends to give a relatively large decrease in mean Gini-gain [106]. The resulting confusion matrix was constructed by using the ‘ggplot2’ package. The model was validated by permutation testing performed in R with 100000 data shuffles.

## Supporting information

S1 Fig Viral load, divergence and diversity analysis in bNAb and no-bNAb individuals. (**A**) Matched viral loads of bNAb (red) and no-bNAb groups (blue) at 6 months post-infection. (**B**) Percentage divergence of autologous viral gp120 sequences from the antigen sequences of ConC, C.ZA.1197MB and CAP45.G3 among 10 bNAb and 7 no-bNAb individuals. (**C**) Diversity analysis of gp160 sequences among 8 bNAb and 5 no-bNAb individuals at 6, 12 and 36 months post-infection were calculated using mean pairwise genetic distances. Significant differences were calculated by Kruskal-Wallis test (n.s. = non-significant).
(PDF)

S2 Fig Fc effector function over time in bNAb and no-bNAb individuals. ADCP (% bead uptake x MFI), ADCD (% C3b deposition x MFI), ADCT (% PKH-26+CFSE+ cells) and ADCC (% Granzyme B) levels were measured against 3 HIV-specific antigens for 13 bNAb individuals (shown in red) and 10 no-bNAb individuals (shown in blue) at 6, 12 and 36 months post-infection. Grey horizontal bars indicate significant comparisons between the groups at single time points (Mann-Whitney U test) and black bars indicate comparisons within each group over time (Kruskal-Wallis test with Tukey´s multiple comparison correction). *<0.05, **<0.001, ***<0.0001. Data are representative of 3 independent experiments. Dotted horizontal lines indicate 3 standard deviations of the mean of the HIV-negative samples.
(PDF)

S3 Fig Broadly neutralizing plasma antibodies mediate Fc effector functions. (**A**) Antibodies that mediate broad neutralization in CAP255 at 3 years post-infection were adsorbed out by ST09 (1gut-mV3 scaffold) as shown by the loss of neutralization against viral isolate Q23.17 (dotted line) compared to unadsorbed IgG (blank solid line). (**B**) Unabsorbed IgG (solid) and absorbed IgG (dashed) were measured for Fc effector functions and significant depletion of these functions is shown as **p<0.001; *p<0.05; **p<0.001 respectively (one-way ANOVA with Tukey correction).
(PDF)

S4 Fig Concordance of Fc functions among bNAb and no-bNAb individuals. (**A**) Spearman´s correlation between the Fc polyfunctionality Z-scores using gp120 ConC and gp140 C.ZA.1197MB where red indicates bNAb and blue no-bNAb individuals. (B) Spearman´s correlation between the Fc polyfunctionality Z-score using gp140 C.ZA.1197MB and neutralization breadth where dotted trend lines are indicated. (**C**) Spearman correlation coefficients between Fc effector functions against gp120 ConC in bNAb individuals (n = 13) and no-bNAb individuals (n = 10) at 6 months post-infection with positive R values shown in purple and negative in green. Color intensity indicates strength of the correlation and significant associations are shown as *p<0.05 and **p<0.001.
(PDF)

S5 Fig Correlations between HIV-specific IgG levels and Fc effector functions. (**A**) Antigen-specific total IgG levels were measured by Luminex with significant differences between groups indicated as *<0.05, **<0.01, ****<0.0001 by Kruskal-Wallis test and Tukey multiple correction. Medians and interquartile ranges are indicated. (**B**) Correlations between gp120 ConC-specific Fc effector functions and gp120 ConC-specific IgG levels (MFI) are shown at 6 months post-infection. Significant Spearman’s correlations are shown in red and ***p<0.001; *p = 0.01. Dotted trend lines are indicated and results are representative of 2 independent experiments.
(PDF)

S6 Fig HIV-specific antigen binding of IgG subclasses in bNAb and no-bNAb individuals. Abundance of IgG1-4 subclasses relative to total antigen specific IgG of bNAb (red) and no-bNAb (blue) individuals shown in columns against 12 HIV antigen shown in rows. Significance between groups was determined by Mann-Whitney U test where *p<0.05.
(PDF)

S7 Fig Gating strategy to define AID-expressing B cells by flow cytometry. **(A**) Representative flow cytometry plots showing PBMCs gated on lymphocytes and single B cells on CD19 + CD3/CD16/CD14. Live B cells were then gated on AID and Ki67. FMO controls for AID and Ki67 as well as an unstimulated and TLR9 stimulated data set are shown. (**B**) Percentage of AID expressing B cells in 6 bNAb, 6 no-bNAb and 4 HIV-negative individuals that were unstimulated (UN) or stimulated with TLR9 for 3 days. (n.s. = non-significant, Kruskal-Wallis test).
(PDF)

S1 Table Participants with and without bNAbs included in this study. (PDF)

S2 Table List of variables significantly associated with bNAb individuals adjusted for multiple comparisons. Significant variables after adjustment by the Benjamini–Hochberg (BH) procedure are shown in bold with their corresponding adjusted p-value shown in italics.
(PDF)
