# Supplementary figures and images for: HIV-specific Fc effector function early in infection predicts the development of broadly neutralizing antibodies
Source: PLoS Pathog. 2018 Apr 9;14(4):e1006987. doi: 10.1371/journal.ppat.1006987 (PMC5908199; doi:10.1371/journal.ppat.1006987)

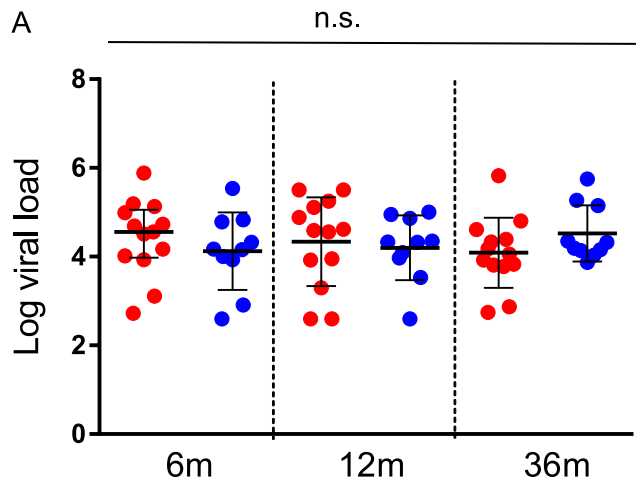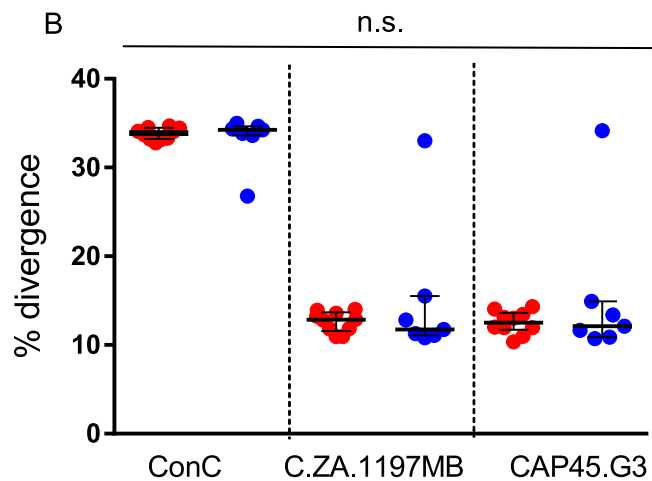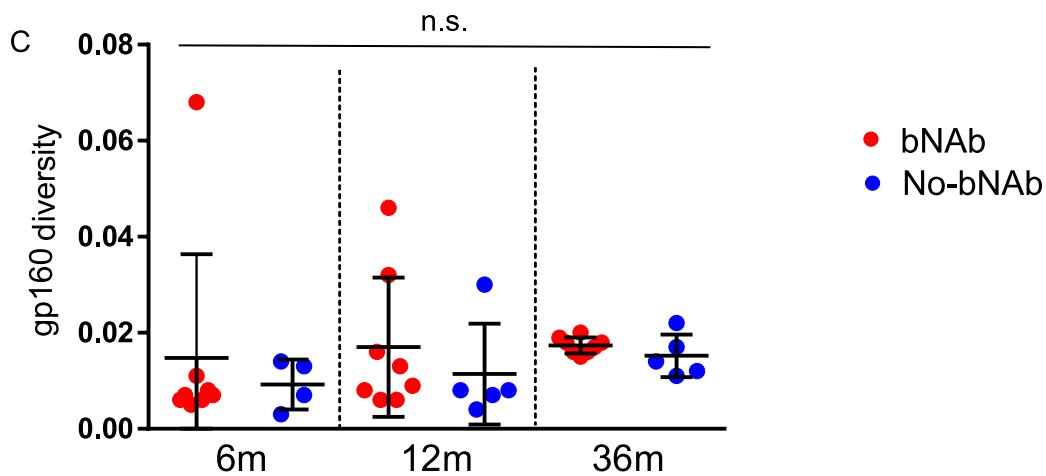

Supplement: S1 Fig — (A) Matched viral loads of bNAb (red) and no-bNAb groups (blue) at 6 months post-infection. (B) Percentage divergence of autologous viral gp120 sequences from the antigen sequences of ConC, C.ZA.1197MB and CAP45.G3 among 10 bNAb and 7 no-bNAb individuals. (C) Diversity analysis of gp160 sequences among 8 bNAb and 5 no-bNAb individuals at 6, 12 and 36 months post-infection were calculated using mean pairwise genetic distances. Significant differences were calculated by Kruskal-Wallis test (n.s. = non-significant). (PDF) [file ppat.1006987.s001.pdf]

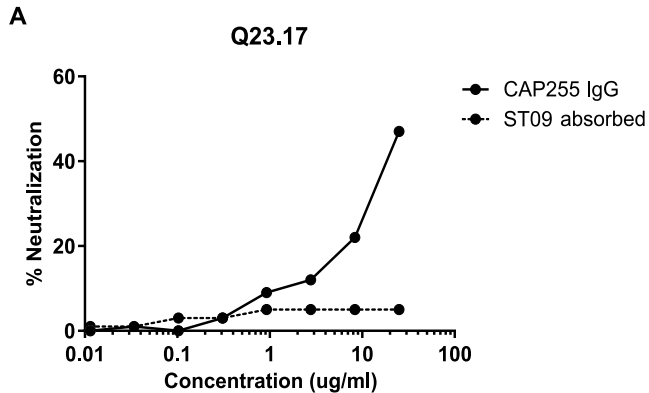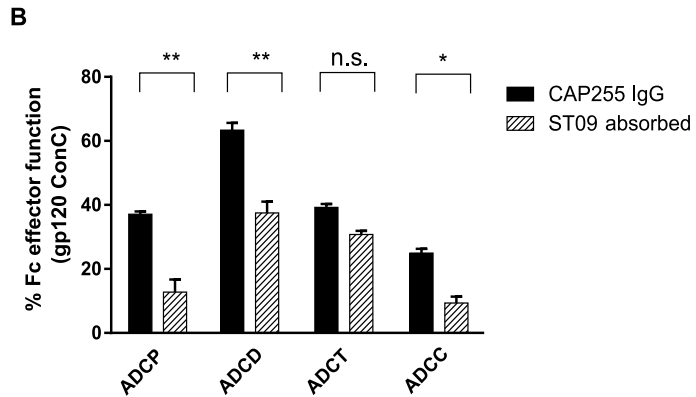

Supplement: S3 Fig — (A) Antibodies that mediate broad neutralization in CAP255 at 3 years post-infection were adsorbed out by ST09 (1gut-mV3 scaffold) as shown by the loss of neutralization against viral isolate Q23.17 (dotted line) compared to unadsorbed IgG (blank solid line). (B) Unabsorbed IgG (solid) and absorbed IgG (dashed) were measured for Fc effector functions and significant depletion of these functions is shown as **p<0.001; *p<0.05; **p<0.001 respectively (one-way ANOVA with Tukey correction). (PDF) [file ppat.1006987.s003.pdf]

A

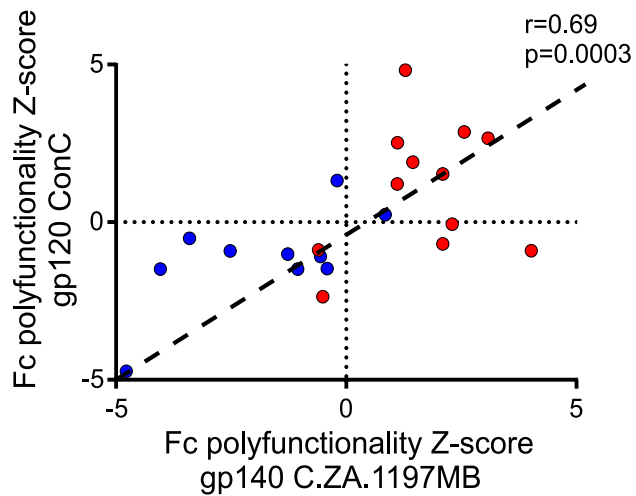

B

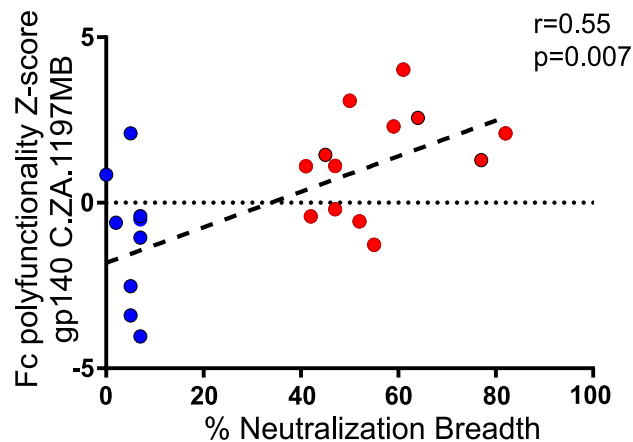

C

bNAbs

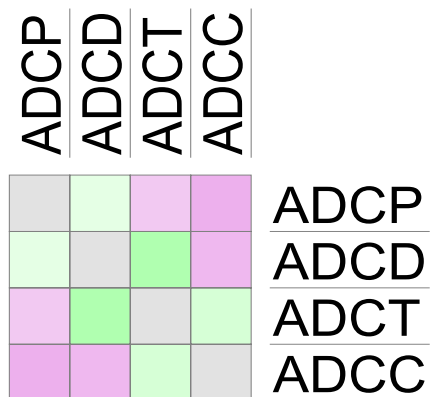

no-bNAbs

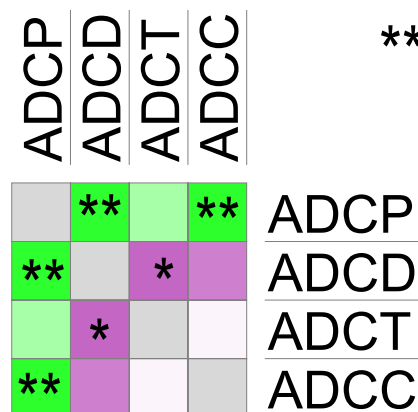

\* &lt; 0.05

\*\* &lt; 0.001

Spearman's correlation coefficient

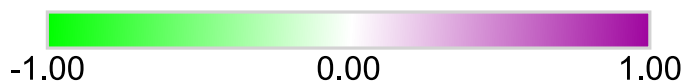

Supplement: S4 Fig — (A) Spearman´s correlation between the Fc polyfunctionality Z-scores using gp120 ConC and gp140 C.ZA.1197MB where red indicates bNAb and blue no-bNAb individuals. (B) Spearman´s correlation between the Fc polyfunctionality Z-score using gp140 C.ZA.1197MB and neutralization breadth where dotted trend lines are indicated. (C) Spearman correlation coefficients between Fc effector functions against gp120 ConC in bNAb individuals (n = 13) and no-bNAb individuals (n = 10) at 6 months post-infection with positive R values shown in purple and negative in green. Color intensity indicates strength of the correlation and significant associations are shown as *p<0.05 and **p<0.001. (PDF) [file ppat.1006987.s004.pdf]

A

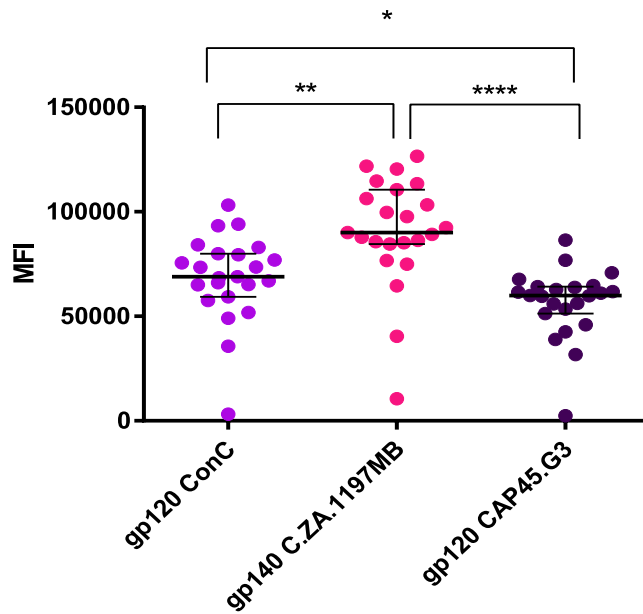

B

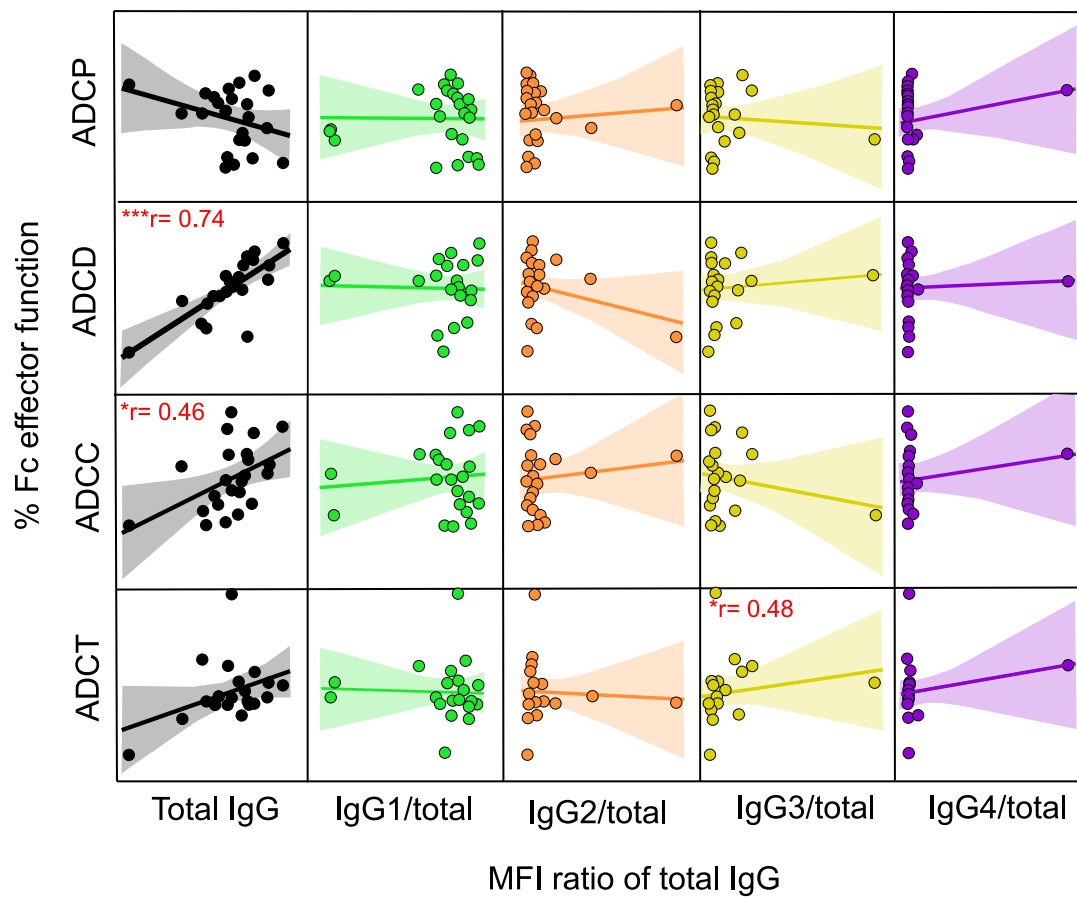

Supplement: S5 Fig — (A) Antigen-specific total IgG levels were measured by Luminex with significant differences between groups indicated as *<0.05, **<0.01, ****<0.0001 by Kruskal-Wallis test and Tukey multiple correction. Medians and interquartile ranges are indicated. (B) Correlations between gp120 ConC-specific Fc effector functions and gp120 ConC-specific IgG levels (MFI) are shown at 6 months post-infection. Significant Spearman’s correlations are shown in red and ***p<0.001; *p = 0.01. Dotted trend lines are indicated and results are representative of 2 independent experiments. (PDF) [file ppat.1006987.s005.pdf]

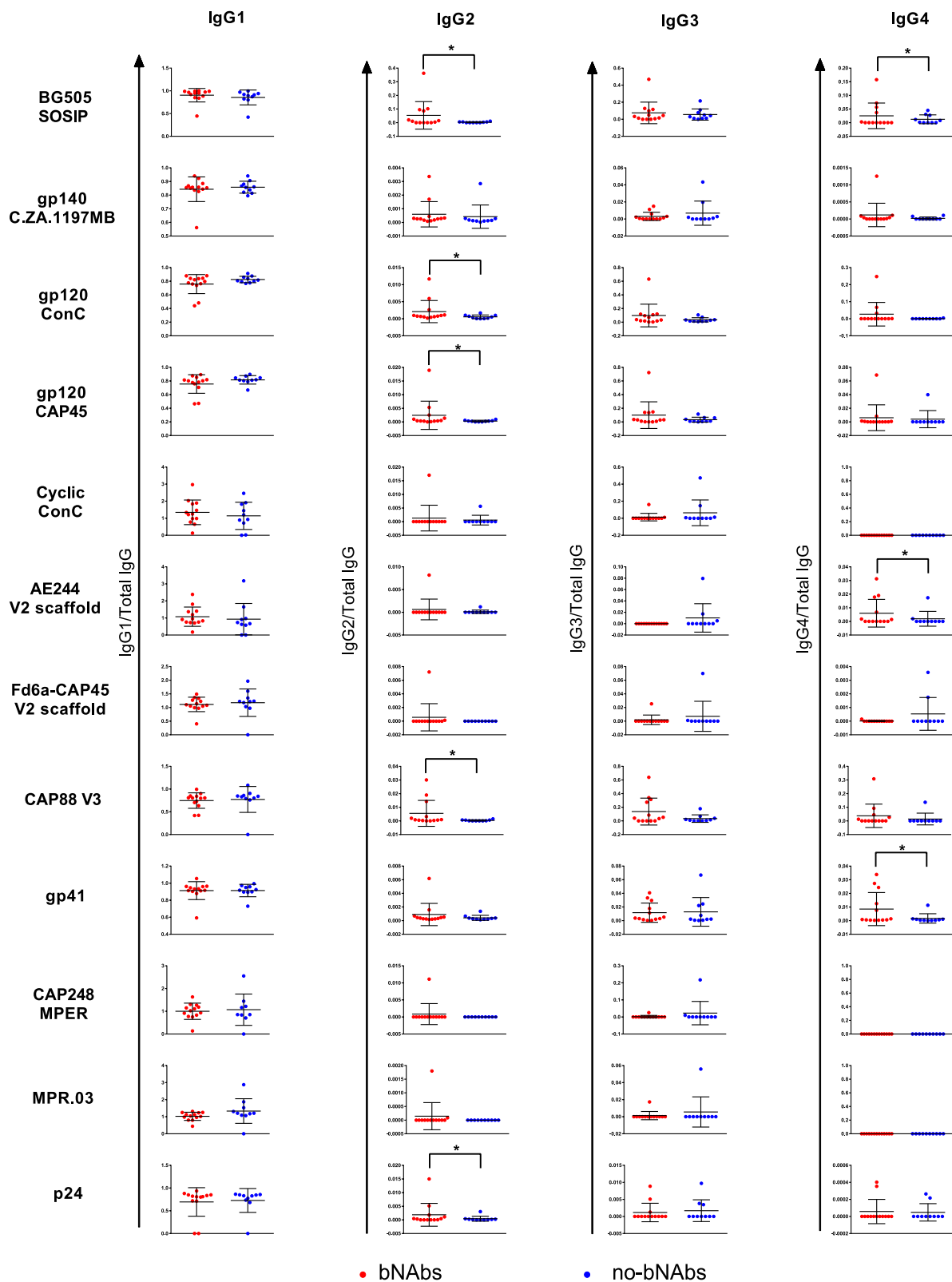

Supplement: S6 Fig — Abundance of IgG1-4 subclasses relative to total antigen specific IgG of bNAb (red) and no-bNAb (blue) individuals shown in columns against 12 HIV antigen shown in rows. Significance between groups was determined by Mann-Whitney U test where *p<0.05. (PDF) [file ppat.1006987.s006.pdf]

**A**

Lymphocytes

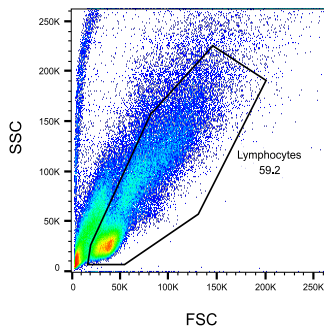

Singlets

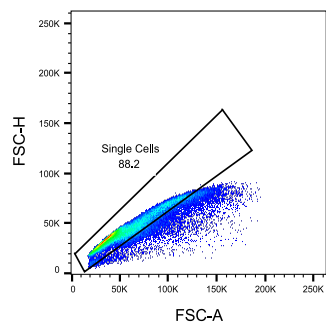

B cells

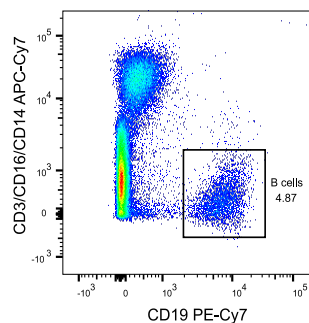

Live

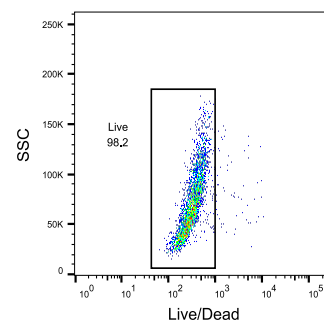

AID-FMO

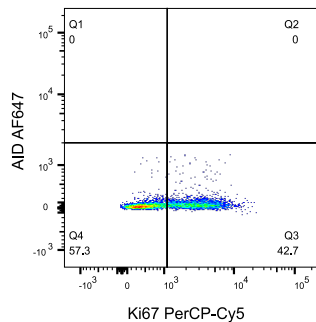

Ki67-FMO

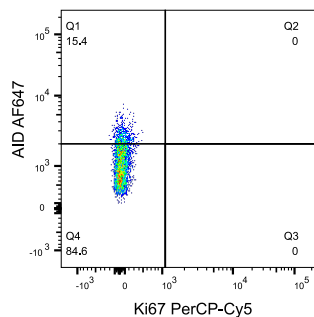

Unstimulated

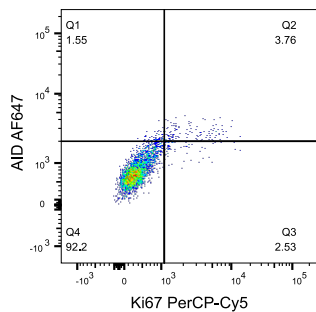

TLR9 Stimulated

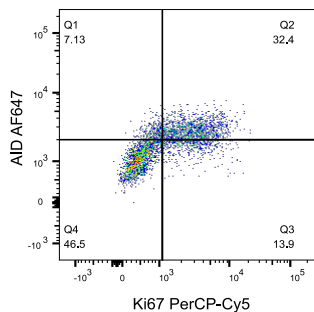**B**

AID+ B cells

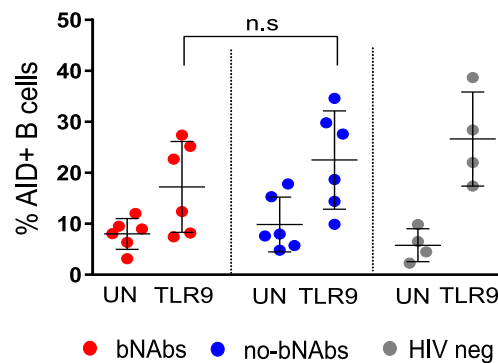

Supplement: S7 Fig — (A) Representative flow cytometry plots showing PBMCs gated on lymphocytes and single B cells on CD19 + CD3/CD16/CD14. Live B cells were then gated on AID and Ki67. FMO controls for AID and Ki67 as well as an unstimulated and TLR9 stimulated data set are shown. (B) Percentage of AID expressing B cells in 6 bNAb, 6 no-bNAb and 4 HIV-negative individuals that were unstimulated (UN) or stimulated with TLR9 for 3 days. (n.s. = non-significant, Kruskal-Wallis test). (PDF) [file ppat.1006987.s007.pdf]
