# Supplementary material for: HIV-specific Fc effector function early in infection predicts the development of broadly neutralizing antibodies
Source: PLoS Pathog. 2018 Apr 9;14(4):e1006987. doi: 10.1371/journal.ppat.1006987 (PMC5908199; doi:10.1371/journal.ppat.1006987)
Supplement: S2 Fig — ADCP (% bead uptake x MFI), ADCD (% C3b deposition x MFI), ADCT (% PKH-26+CFSE+ cells) and ADCC (% Granzyme B) levels were measured against 3 HIV-specific antigens for 13 bNAb individuals (shown in red) and 10 no-bNAb individuals (shown in blue) at 6, 12 and 36 months post-infection. Grey horizontal bars indicate significant comparisons between the groups at single time points (Mann-Whitney U test) and black bars indicate comparisons within each group over time (Kruskal-Wallis test with Tukey´s multiple comparison correction). *<0.05, **<0.001, ***<0.0001. Data are representative of 3 independent experiments. Dotted horizontal lines indicate 3 standard deviations of the mean of the HIV-negative samples. (PDF) [file ppat.1006987.s002.pdf]

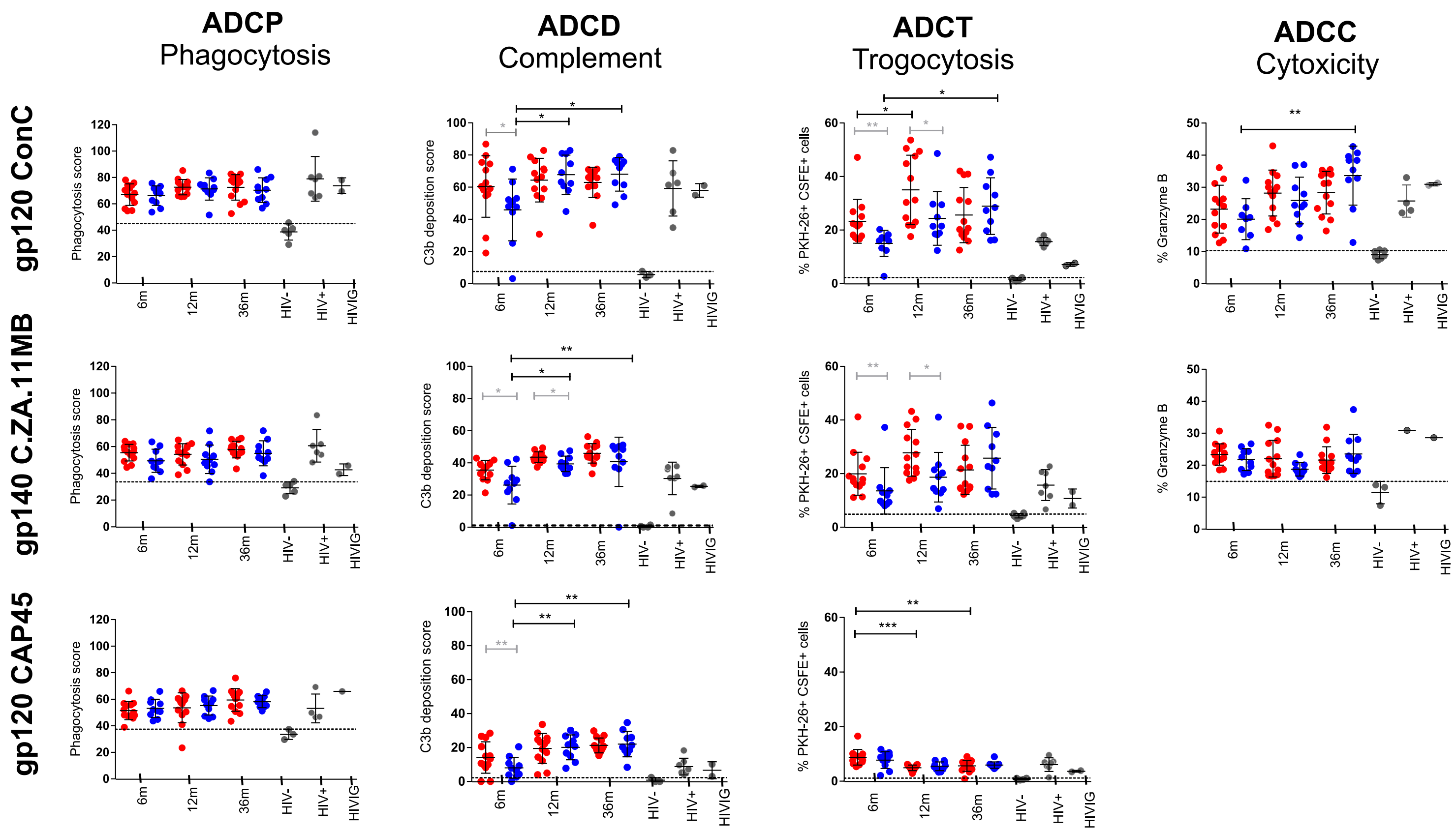
ADCP
Phagocytosis
ADCD
Complement
ADCT
Trogocytosis
ADCC
Cytoxicity
gp120 ConC
gp140 C.ZA.11MB
gp120 CAP45
Phagocytosis score
C3b deposition score
% PKH-26+ CSFE+ cells
% Granzyme B
6m
12m
36m
HIV-
HIV+
HIVIG
