# Supplementary material for: HIV-specific Fc effector function early in infection predicts the development of broadly neutralizing antibodies
Source: PLoS Pathog. 2018 Apr 9;14(4):e1006987. doi: 10.1371/journal.ppat.1006987 (PMC5908199; doi:10.1371/journal.ppat.1006987)
Supplement: S1 Table — (PDF) [file ppat.1006987.s008.pdf]

S1 Table. Participants with and without bNAbs included in this study
Table A. Participants with bNAbs

Participants	Viral load	CD4 (cells/uL)	% Neutralization breadth (3 years)
CAP257	14800	528.0	82.0
CAP256	750000	500.0	77.0
CAP312	10400	692.0	64.0
CAP287	36300	277.0	61.0
CAP248	1290	355.0	59.0
CAP292	134000	492.0	55.0
CAP177	32300	426.0	52.0
CAP341	521	322.0	50.0
CAP206	156000	267.0	47.0
CAP255	49200	415.0	47.0
CAP357	8490	418.0	45.0
CAP8	98400	343.0	42.0
CAP288	53300	295.0	41.0
Median	***36300.0***	***415.0***	***52.0***

Table B. Participants without bNAbs

Participants	Viral load	CD4 (cells/uL)	% Neutralization breadth (3 years)
CAP188	66900	427.0	11.0
CAP228	817	816.0	7.0
CAP229	8470	607.0	7.0
CAP265	400	954.0	7.0
CAP268	14800	668.0	7.0
CAP225	59700	496.0	5.0
CAP237	9960	492.0	5.0
CAP271	14400	463.0	5.0
CAP200	349000	440.0	2.0
CAP88	21100	864.0	0.0
Median	***14600.0***	***551.5***	***6.0***

Viral load and CD4 count at 6 months post-infection and % neutralization breadth against a 44 virus panel at 3 years post-infection.
