# Supplementary material for: HIV-specific Fc effector function early in infection predicts the development of broadly neutralizing antibodies
Source: PLoS Pathog. 2018 Apr 9;14(4):e1006987. doi: 10.1371/journal.ppat.1006987 (PMC5908199; doi:10.1371/journal.ppat.1006987)
Supplement: S2 Table — Significant variables after adjustment by the Benjamini–Hochberg (BH) procedure are shown in bold with their corresponding adjusted p-value shown in italics. (PDF) [file ppat.1006987.s009.pdf]

Table S2. List of variables significantly associated with bNAb individuals adjusted for multiple comparisons

Variable	P value	BH critical value	Adjusted P value	Spearmans coefficient
**gp120 ConC FcγRIIIa-V158**	0.0003	0.0017	*0.0087*	0.69
**gp120 ConC FcγRIIb**	0.0004	0.0034	*0.0058*	0.67
**gp120 CAP45 FcγRIIIa-V158**	0.0006	0.0052	*0.0058*	0.66
**IgG total gp120 ConC**	0.0015	0.0069	*0.0109*	0.62
**gp140 Fc polyfunction**	0.0027	0.0086	*0.0157*	0.60
**CD4 count**	0.0031	0.0103	*0.0150*	-0.59
**gp120 ConC Fc polyfunction**	0.0036	0.0121	*0.0149*	0.58
**gp120 CAP45 FcγRIIIb NA2**	0.0058	0.0138	*0.0210*	0.56
**gp120 ConC C1q**	0.0059	0.0155	*0.0190*	0.56
**CXCL13**	0.0075	0.0172	*0.0218*	0.54
**gp120 ConC FcγRIIIb NA2**	0.0075	0.0190	*0.0198*	0.54
**gp120 ConC FcγRIIb**	0.0083	0.0207	*0.0201*	0.54
**gp120 CAP45 FcγRIIIa-F158**	0.0094	0.0224	*0.0210*	0.53
**gp120 ConC FcγRIIIa-F158**	0.0118	0.0241	*0.0244*	0.52
**gp120 CAP45 FcγRIIa-H131**	0.0153	0.0259	*0.0296*	0.50
**gp120 ConC subclass diversity**	0.0178	0.0276	*0.0323*	0.49
**IgG total gp120 CAP45**	0.0262	0.0293	*0.0447*	0.46
gp120 ConC FcγRIIa-R131	0.0312	0.0310	0.0503	0.45
CAP45 C1q	0.0366	0.0328	0.0559	0.44
IgG total gp140	0.0443	0.0345	0.0642	0.42
gp120 CAP45 FcγRIIa-R131	0.0642	0.0362	0.0887	0.39
Total IgG1	0.0752	0.0379	0.0991	0.38
gp140 subclass diversity	0.0817	0.0397	0.1030	0.37
gp120 ConC FcγRIIa-H131	0.0941	0.0414	0.1137	0.36
gp120 CAP45 subclass diversity	0.1081	0.0431	0.1254	0.34
Total IgG	0.1583	0.0448	0.1766	0.30
Total IgG3	0.1781	0.0466	0.1913	-0.29
gp120 CAP45 Fc polyfunction	0.2227	0.0483	0.2307	0.26
Log viral load	0.3641	0.0500	0.3641	0.20

Significant variables after adjustment by the Benjamini–Hochberg (BH) procedure are shown in bold with their corresponding adjusted p-value shown in italics.
